# Impact of Prehospital Lung Ultrasound on Diagnostic Precision and Hospital Transport in Patients with Dyspnea and Respiratory Failure: A Retrospective Comparative Analysis

**DOI:** 10.3390/diagnostics16091297

**Published:** 2026-04-26

**Authors:** Damian Kowalczyk, Mikołaj Tyczyński

**Affiliations:** 1 Emergency Medical Service, Healthcare Center “ZOZ Legionowo”, 4 General Józef Sowiński Street, 05-120 Legionowo, Poland; 2 Department of Emergency Medical Service, Academy of Medical, Applied and Holistic Sciences, 02-304 Warsaw, Poland

**Keywords:** prehospital lung ultrasound, point-of-care ultrasound, lung ultrasound, LUS, dyspnea, emergency medicine, prehospital diagnosis, emergency medical services

## Abstract

**Background:** Dyspnea is a common reason for emergency medical service (EMS) interventions and is associated with a substantial risk of severe clinical course, complications, and hospital admission. Its differential diagnosis in the prehospital setting remains challenging due to the limited availability of imaging modalities. Point-of-care ultrasound (POCUS), including lung ultrasound (LUS), is a rapid, field-applicable technique recommended in numerous acute respiratory diagnostic scenarios. **Objective**: To evaluate the use of lung ultrasound in the prehospital setting and its association with the precision of diagnoses related to respiratory failure, the frequency of transport to the emergency department (ED) among patients presenting with dyspnea/respiratory failure, and to characterize the profile of sonographic findings with their correlation to clinical diagnostic categories. Additionally, transport rates in the study population were compared with aggregated regional data for the Masovian Voivodeship (excluding the analyzed county). **Methods**: A retrospective observational study was conducted on EMS interventions performed between 01 January 2025 and 30 June 2025 in Legionowo County (*N* = 353). The analysis included ICD-10 codes assigned in prehospital documentation (one primary code and up to two additional codes) in patients presenting with dyspnea and/or respiratory failure, the performance of ultrasound examination, and resulting LUS findings (absence of pleural sliding and/or lung point; B-lines; consolidations; C-lines; pleural effusion). Descriptive analyses, frequency comparison tests (χ^2^/Fisher), estimation of relative risk (RR) with 95% confidence intervals (CI), and agreement analysis using Cohen’s kappa coefficient (κ) between etiological categories derived from ICD-10 codes and those inferred from LUS profiles were performed (κ with 95% CI estimated using bootstrap resampling). The study was reported in accordance with the STROBE guidelines for observational studies. Additionally, the distribution of ICD-10 coding and the proportion of hospital transports across the entire Masovian Voivodeship were compared with those observed in the analyzed area. **Results**: Ultrasound examination was performed in 72/353 (20.4%) EMS interventions; transport to the emergency department occurred in 239/353 (67.7%) cases. The most frequent clinical categories based on ICD-10 codes were: general/symptom-based 182/353 (51.6%), inflammatory 77/353 (21.8%), obstructive 66/353 (18.7%), and cardiological 20/353 (5.7%). Among abnormal LUS findings, the most common were B-lines (43/72; 61.4%) and consolidations (29/72; 41.4%). Consolidations were strongly associated with the inflammatory category (OR 9.72; *p* < 0.001), whereas B-lines were associated with the cardiological category (OR 23.41; *p* = 0.0011) among cases in which LUS was performed. Ultrasound use was associated with a higher frequency of assigning at least one targeted (non-symptom-based) diagnosis within ICD coding: 53/72 (73.6%) vs. 111/278 (39.9%), RR 1.84 (95% CI 1.51–2.25; *p* < 0.001). Agreement between the ICD-10 etiological category (inflammatory/cardiological/obstructive/other) and the category inferred from the LUS profile was moderate: κ = 0.36 (95% CI 0.21–0.51), with an observed agreement of 54.2%. Compared with aggregated regional data (Masovian Voivodeship excluding the analyzed county), the overall transport rate for comparable ICD-10 codes was lower in the study unit: 279/409 (68.2%) vs. 11,351/13,785 (82.3%), RR 0.83 (95% CI 0.78–0.89; *p* < 0.001). The largest differences were observed for dyspnea (R06.0: 72.9% vs. 88.2%; RR 0.83) and obstructive codes (J44/J45/J46 combined: 43.1% vs. 67.0%; RR 0.64). **Conclusions**: In this retrospective analysis, an EMS unit with systematically implemented ultrasound demonstrated a lower frequency of hospital transport for selected dyspnea/respiratory failure codes compared with regional data and greater precision in ICD-10 diagnostic coding in cases where ultrasound was performed. The profile of LUS findings correlated with clinical categories in a manner consistent with existing literature.

## 1. Introduction

Dyspnea and broadly defined respiratory failure represent one of the most clinically significant reasons for emergency medical service (EMS) interventions [1]. In large cohorts, these conditions account for a substantial proportion of EMS and emergency department (ED) contacts, and a considerable subset of patients ultimately requires hospital admission or intensive care management [2].

From the perspective of prehospital practice, a key challenge is the rapid differentiation of the underlying causes of dyspnea, including obstructive conditions (chronic obstructive pulmonary disease [COPD] or asthma), infectious etiologies (such as pneumonia), cardiogenic causes (acute pulmonary edema or decompensated heart failure), and pleural conditions (e.g., pneumothorax or pleural effusion). In the prehospital setting, this differentiation is particularly difficult because clinical manifestations—such as tachypnea, hypoxemia, and auscultatory abnormalities—often overlap between disease entities [3]. As a result, clinical assessment alone frequently does not allow reliable identification of the underlying cause without additional diagnostic imaging, including lung ultrasound.

Point-of-care ultrasound has become an important tool supporting rapid diagnostic and therapeutic decision-making in emergency medicine [4]. In parallel, its application in the prehospital setting has expanded significantly due to the miniaturization of ultrasound devices, the growing availability of dedicated training programs, and an increasing body of scientific evidence supporting its use [5]. Among the various POCUS applications, lung ultrasound is particularly attractive for the differential diagnosis of acute dyspnea because it relies on the recognition of reproducible ultrasound artifacts—such as A-lines, B-lines, consolidations, and pneumothorax signs—which are closely associated with specific pathophysiological conditions [6].

Previous studies have demonstrated the high diagnostic utility of selected LUS findings. B-lines have shown high sensitivity and specificity in the diagnosis of acute pulmonary edema [4]. Pneumothorax signs, including the lung point, are highly specific for the diagnosis of pneumothorax [6]. Furthermore, ultrasound is highly sensitive in detecting pleural effusion, often outperforming conventional chest radiography [7]. For pneumonia, meta-analyses indicate high diagnostic accuracy of LUS, frequently demonstrating higher sensitivity than chest radiography while maintaining comparable or high specificity depending on the studied population [8].

In the prehospital environment, the available literature suggests that LUS examinations are feasible after relatively short training programs and that POCUS may influence both therapeutic and transport decisions [9]. In acute heart failure, several studies have also demonstrated improved diagnostic accuracy of prehospital assessments when LUS is incorporated into the diagnostic process [10]. Despite these promising findings, system-level analyses evaluating the relationship between ultrasound availability and use in EMS units and clinically relevant decision endpoints—such as hospital transport rates—remain limited.

In this context, the present study aims to simultaneously:

(1) Describe the actual frequency of LUS use among patients presenting with dyspnea or respiratory failure in the prehospital setting;
(2) Characterize the spectrum of observed sonographic findings and their correlation with diagnostic categories;
(3) Evaluate whether the use of ultrasound is associated with more targeted ICD-10 diagnostic coding;
(4) Compare hospital transport rates in the studied EMS unit with regional data obtained from the national EMS monitoring registry.

## 2. Materials and Methods

### 2.1. Study Design

This study was designed as a retrospective observational analysis of emergency medical service (EMS) interventions performed between 1 January 2025 and 30 June 2025 in Legionowo County, Poland. The aim of the study was to evaluate the role of lung ultrasound (LUS) in prehospital diagnostics among patients presenting with dyspnea or respiratory failure, including its association with etiology-oriented diagnostic coding, the frequency of hospital transport, and the correlation between sonographic findings and clinical diagnostic categories. Although diagnostic accuracy was not directly assessed, the observed shift toward more etiology-oriented ICD-10 coding suggests that LUS may support more structured clinical reasoning in prehospital settings. The study additionally included a comparative analysis with aggregated regional data obtained from the National Emergency Medical Services Monitoring Centre, covering the same ICD-10 categories for the Masovian Voivodeship, excluding Legionowo County. Legionowo County is an urban–suburban region located in central Poland, with a population of approximately 125,000 inhabitants. The Masovian Voivodeship, representing the largest administrative region in Poland, includes approximately 5.4 million inhabitants. Population data were obtained from Statistics Poland (Główny Urząd Statystyczny, GUS).

### 2.2. Participants

The study population consisted of patients attended by EMS teams in Legionowo County during the study period. Patients were eligible for inclusion if the EMS medical documentation contained at least one ICD-10 code corresponding to dyspnea, respiratory failure, or related respiratory conditions. According to the Polish EMS documentation system, the team leader is required to assign at least one ICD-10 code and up to three codes (one primary diagnosis and up to two additional diagnoses). All recorded codes were included in the analysis. Each patient was counted once in the study population, while all assigned ICD-10 codes were considered during the classification of clinical categories. No participant enrollment or prospective consent procedure was applied because the study was retrospective and based on de-identified routine EMS documentation.

### 2.3. Inclusion Criteria

Patients were included if the EMS intervention met the following criteria: intervention performed by an EMS team, intervention date between 1 January 2025 and 30 June 2025, location of intervention within Legionowo County, and the presence of at least one predefined ICD-10 code related to dyspnea, respiratory failure, or associated respiratory conditions. The ICD-10 codes were grouped into predefined clinical categories, as presented in Table 1.

Heart failure codes such as I50 were not included in the predefined cardiological category because the study focused on ICD-10 codes directly reflecting respiratory failure, dyspnea, or respiratory system dysfunction. In routine prehospital practice, I50 may also be assigned to patients with predominantly circulatory or systemic manifestations of heart failure without relevant acute respiratory impairment, which could reduce the specificity of the respiratory-focused analytical framework applied in the present study.

### 2.4. Data Collection

Data were extracted retrospectively from prehospital EMS medical documentation, including emergency medical intervention records. The following variables were collected: assigned ICD-10 diagnostic codes, performance of lung ultrasound (LUS) during EMS intervention, sonographic findings observed in LUS examination, decision regarding transport to the emergency department and final prehospital ICD-10 coding. The full dataset of patients from Legionowo County, including assigned ICD-10 codes and corresponding lung ultrasound findings, is presented in Supplementary Table S2.

### 2.5. Lung Ultrasound Assessment

In cases where lung ultrasound was performed, the presence of the following sonographic findings was recorded: absence of pleural sliding and/or lung point (suggestive of pneumothorax), B-lines, pulmonary consolidations, subpleural consolidations (C-line-type artifacts), and pleural effusion. The distribution of sonographic findings was analyzed and correlated with the ICD-10 clinical categories in order to determine which ultrasound patterns were most frequently associated with specific disease groups. All lung ultrasound examinations were performed using the same ultrasound device Philips Lumify (Philips Healthcare, Amsterdam, The Netherlands) equipped with a curvilinear (convex) transducer, as a standardized ultrasound system was implemented across all EMS teams included in the study. Lung ultrasound was used as an adjunct to the standard physical examination. The decision to perform the examination, as well as its timing during the prehospital encounter, was left to the clinical judgment of the EMS team leader (paramedic), without predefined criteria for its use and no rigid protocol mandating ultrasound use was applied. For interpretative purposes, consolidations and subpleural consolidations were considered predominantly inflammatory patterns, B-lines without consolidation as predominantly cardiological patterns, pneumothorax signs as non-inflammatory pleural pathology, and normal aeration profiles as compatible with obstructive presentations in the appropriate clinical context. For clarity, the relationship between lung ultrasound findings and clinical diagnostic categories is summarized in Table 2.

### 2.6. EMS Ultrasound Implementation

In the EMS unit operating in Legionowo County, lung ultrasound has been systematically implemented across all EMS teams since 2024. Each EMS team is equipped with a portable ultrasound device, and all team leaders have completed at least one certified training course in prehospital ultrasound diagnostics. This organizational structure allows the routine use of LUS during prehospital interventions. All EMS team leaders participating in the study completed the same internal ultrasound training program, consisting of a 6 h basic course and 8 h advanced course delivered by experienced practitioners. In addition, all operators had prior clinical experience in emergency medical services and routinely performed lung ultrasound examinations in daily practice following the implementation of ultrasound in 2024. The training focused, among others, on the differential diagnosis of respiratory failure using ultrasound, based on the BLUE protocol. In the studied EMS unit, lung ultrasound was performed by paramedics serving as EMS team leaders. Ultrasound interpretation was performed on scene by EMS personnel as part of routine clinical care. No formal centralized image archiving, cloud transmission, or external quality assurance review process was implemented during the study period. Lung ultrasound had been systematically implemented across all EMS teams since 2024, preceding the study period. During the study period, the EMS system in Legionowo County consisted of five EMS teams. All teams had continuous 24 h access to the same ultrasound devices and transducer configuration.

### 2.7. Regional EMS Ultrasound Availability

To assess the availability of ultrasound in other EMS units within the Masovian Voivodeship, official inquiries were sent to all EMS service providers in the region. The following institutions were contacted:

- Warsaw EMS and Medical Transport Service (“Meditrans”)
- Radom EMS Service
- EMS and Medical Transport Service in Siedlce
- EMS Service in Płock
- EMS Service “Meditrans-Ostrołęka”
- Nowy Dwór Mazowiecki Medical Center
- Public Healthcare Complex in Przasnysz
- Mazovian Specialist Hospital in Radom
- Bródnowski Hospital in Warsaw
- EMS Service in Błonie

Responses indicated that:

- Some EMS providers do not equip their EMS teams with ultrasound devices,
- In certain services, ultrasound is available only in physician-staffed advanced EMS teams,
- Several EMS providers did not respond to the inquiry.

Responses indicated that some EMS providers do not equip their EMS teams with ultrasound devices (2/10; 20.0%), in certain services, ultrasound is available only in physician-staffed advanced EMS teams (1/10; 10.0%), and several EMS providers did not respond to the inquiry (7/10; 70.0%). These findings suggest that systematic implementation of ultrasound across all EMS teams remains uncommon in the region.

### 2.8. Regional Comparison Data

Aggregated data for the same ICD-10 categories were obtained from the National Emergency Medical Services Monitoring Centre for the Masovian Voivodeship, excluding Legionowo County. These data included: the number of EMS interventions, assigned ICD-10 codes and the proportion of hospital transports. This allowed comparison between the study EMS unit with systematic ultrasound availability and the regional EMS system, where ultrasound availability is limited or inconsistent. Detailed aggregated regional data obtained from the National Emergency Medical Services Monitoring Centre are provided in Supplementary Table S1.

### 2.9. Statistical Analysis

Continuous variables were assessed for normality using the Shapiro–Wilk test. As the distribution of continuous variables differed from normal, they were presented as the median and interquartile range (IQR), from the lower quartile (LQ, 25%) to the upper quartile (UQ, 75%). Categorical variables were presented as absolute numbers and percentages. Differences between categorical variables were assessed using the Chi-square (χ^2^) test or Fisher’s exact test when appropriate. The association between sonographic findings in lung ultrasound (LUS) and clinical categories based on ICD-10 codes was assessed using odds ratios (ORs) with corresponding 95% confidence intervals (CI) [11]. Relative risk (RR) with 95% CI was additionally calculated for selected comparisons, including the frequency of targeted diagnoses and transport to the emergency department. Agreement between etiological categories derived from ICD-10 codes and categories inferred from lung ultrasound findings was assessed using Cohen’s kappa coefficient (κ). The result was expressed as κ with a 95% confidence interval (CI) estimated using bootstrap resampling. Interpretation of the kappa coefficient followed the recommendations published by McHugh et al. [12]. Comparative analysis was additionally performed between the study population (Legionowo County) and aggregated regional data obtained from the National Emergency Medical Services Monitoring Centre for the Masovian Voivodeship (excluding Legionowo County), including the frequency of hospital transport for comparable ICD-10 categories. All statistical tests were two-sided, and a *p*-value < 0.05 was considered statistically significant. Statistical analyses were performed using R software, version 4.3.1 (R Foundation for Statistical Computing, Vienna, Austria). The kappa coefficient was calculated using Statistica 13.1 software (TIBCO Software Inc., Palo Alto, CA, USA). The study was reported in accordance with the STROBE guidelines for observational studies [13]. No a priori sample size or power calculation was performed because the study was retrospective and based on all eligible EMS interventions recorded during the predefined study period.

## 3. Results

### 3.1. Study Population

**Figure 1:**
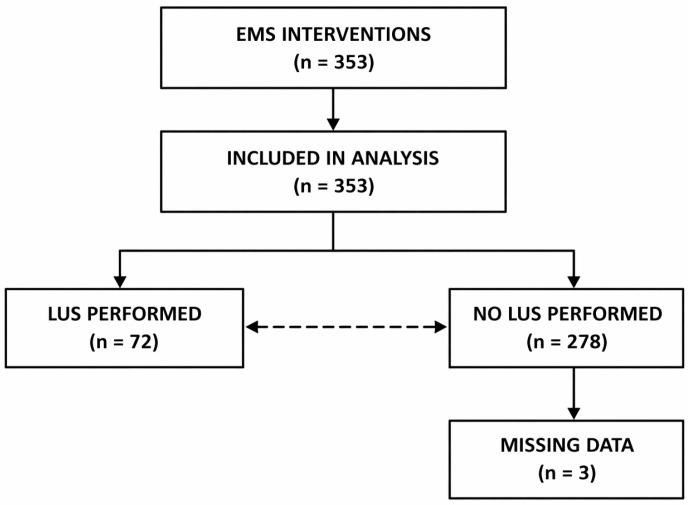
Flow diagram of patient inclusion and lung ultrasound (LUS) utilization in the study population.

A flow diagram of patient inclusion and lung ultrasound (LUS) utilization is presented in Figure 1. The diagram summarizes the distribution of patients according to ultrasound utilization.

During the study period, 353 EMS interventions met the predefined inclusion criteria in the analyzed county (Table 3). In 176/353 cases (49.9%), one ICD-10 code was assigned, in 134/353 (38.0%) two codes were recorded, and in 43/353 (12.2%) three diagnostic codes were documented. Transport to the emergency department (ED) occurred in 239/353 interventions (67.7%). Lung ultrasound was performed in 72/353 interventions (20.4%), while no ultrasound was documented in 278/353 cases (78.8%). Information regarding ultrasound performance was missing in 3/353 cases (0.8%). The frequency of ultrasound use differed between diagnostic categories. The complete dataset underlying the analysis is available in the Supplementary Materials (Supplementary Table S2).

### 3.2. Distribution of Clinical Categories

The majority of cases were classified within the general/symptom-based category, followed by inflammatory and obstructive conditions, whereas cardiological and neoplastic categories were less frequent (Table 4). The highest proportion of hospital transport was observed in the cardiological category. The largest group of cases belonged to the general/symptom-based category.

### 3.3. Lung Ultrasound Findings

Among patients in whom lung ultrasound was performed, the most frequent abnormal findings were B-lines and pulmonary consolidations, followed by subpleural consolidations. Pleural effusion and pneumothorax signs were less common. In 16/72 examinations, no abnormal findings were recorded, corresponding to a normal aeration profile (A-profile) (Table 5).

### 3.4. Association Between LUS Findings and Diagnostic Categories

Within the subgroup of patients who underwent LUS examination, pulmonary consolidations were strongly associated with the inflammatory category (OR 9.72; *p* < 0.0001), whereas B-lines were strongly associated with the cardiological category (OR 23.41; *p* = 0.0011).

### 3.5. Impact of Ultrasound on Diagnostic Precision

The proportion of etiology-oriented diagnoses, defined as the presence of at least one non-symptom-based ICD-10 code among the assigned diagnoses, was 165/353 (46.7%) in the overall study population. Analyses of ultrasound-related diagnostic precision were restricted to cases with documented ultrasound status. This proportion was significantly higher in cases in which ultrasound was performed than in those without documented ultrasound use (53/72 [73.6%] vs. 111/278 [39.9%]; RR 1.84, 95% CI 1.51–2.25; *p* < 0.001) (Table 6). Similarly, assignment of a specific primary ICD-10 diagnosis was more frequent when ultrasound was performed (41/72 [56.9%] vs. 104/278 [37.4%]; RR 1.52, 95% CI 1.18–1.96; *p* = 0.004).

### 3.6. Agreement Between LUS Profile and ICD-10 Etiological Category

Agreement between etiological categories derived from ICD-10 coding (inflammatory/cardiological/obstructive/other) and those inferred from the LUS profile (pneumothorax → other; consolidations or subpleural consolidations → inflammatory; B-lines without consolidation → cardiological; normal aeration profile → obstructive; remaining cases → other) was moderate.

The observed agreement was 54.2%, with κ = 0.36 (95% CI 0.21–0.51). In the subgroup of cases in which ICD coding indicated a clearly defined etiology (excluding the “other” category), the agreement increased to κ = 0.46 (95% CI 0.22–0.66). Among cases classified as “other” according to ICD-10 coding within the ultrasound group (*n* = 21), LUS suggested one of three specific etiological categories (inflammatory, cardiological, or obstructive) in 17/21 cases (81%).

### 3.7. Regional Comparison of Hospital Transport Rates

According to data obtained from the National Emergency Medical Services Monitoring Centre, a total of 204,823 EMS interventions were recorded in the Masovian Voivodeship (excluding the analyzed county) during the study period. The total number of occurrences of the analyzed ICD-10 codes was 14,469, of which 11,906 (82.3%) resulted in hospital transport. For comparable ICD-10 codes present in both datasets, the overall transport rate was lower in the analyzed EMS unit than in the regional dataset (279/409 [68.2%] vs. 11,351/13,785 [82.3%]; RR 0.83, 95% CI 0.78–0.89; *p* < 0.001) (Table 7 and Table 8).

Similar differences were observed for symptom-based and non-specific respiratory codes (R06.* and J96.*), as well as for obstructive airway disease codes (J44/J45/J46) and for R06.0 (dyspnea) (Table 9).

## 4. Discussion

The findings of the present study contribute to the growing body of evidence suggesting that lung ultrasound performed in the prehospital setting may provide meaningful diagnostic support in patients with acute dyspnea and respiratory failure. In the analyzed population, the use of LUS was associated with a higher proportion of etiology-oriented diagnoses and a lower rate of hospital transport compared with regional data, suggesting that systematic implementation of ultrasound in emergency medical services may influence diagnostic reasoning and clinical decision-making.

Existing literature indicates that the use of point-of-care ultrasound (POCUS) in the assessment of acute dyspnea may assist emergency medical teams in directing etiological diagnosis in situations where access to advanced imaging is limited. Meta-analyses addressing the prehospital use of ultrasound in acute respiratory failure suggest that POCUS may improve diagnostic accuracy in the prehospital environment. At the same time, authors of these analyses emphasize that the overall quality of evidence remains limited in several areas and highlight the need for further studies with higher methodological rigor [1].

The results of the present study are consistent with these observations. In the analyzed cohort, the performance of lung ultrasound was associated with a significantly higher proportion of etiology-oriented diagnoses (RR 1.84), suggesting that LUS may support diagnostic reasoning by paramedics already at the prehospital stage. Importantly, the lack of formal image archiving and external quality assurance reflects real-world prehospital practice, where point-of-care ultrasound is primarily used as a bedside decision-support tool rather than a fully standardized imaging modality. While this may limit internal validation of image interpretation, it increases the external validity of the study and reflects actual clinical conditions in EMS systems.

Furthermore, analysis of ultrasound findings demonstrated characteristic relationships between specific sonographic patterns and clinical diagnostic categories. B-lines were strongly associated with the cardiological category, while pulmonary consolidations were strongly associated with inflammatory diagnoses. These observations are consistent with the current understanding of lung ultrasound pathophysiology and with findings reported in hospital-based clinical studies [4].

Mechanistically, the potential impact of LUS on decisions regarding hospital transport may occur through several pathways. First, lung ultrasound may facilitate earlier recognition of conditions requiring urgent hospitalization, such as acute cardiogenic pulmonary edema, where a predominant B-line profile represents a characteristic sonographic pattern. Second, the absence of interstitial congestion or pleural effusion with a dominant A-profile may support the diagnosis of obstructive causes of dyspnea, such as exacerbation of chronic obstructive pulmonary disease or asthma. In clinically stable patients, this information may help avoid precautionary hospital transport. Third, earlier etiological clarification of dyspnea may enable more targeted prehospital treatment, potentially improving patient condition before hospital admission [14].

Previous studies investigating prehospital diagnosis of acute heart failure have demonstrated that lung ultrasound may improve diagnostic accuracy and influence therapeutic decision-making in emergency medical services [15]. In the present study, the observed association between B-lines and cardiologic diagnoses further supports the utility of this sonographic pattern in identifying pulmonary edema in the prehospital environment [16].

The obstructive component of the analysis is particularly relevant in the context of the study objective, which included assessment of hospital transport rates. Previous studies have demonstrated the feasibility of implementing POCUS in the prehospital management of patients with COPD exacerbations. Improved clinical assessment supported by ultrasound may contribute to more appropriate decision-making regarding patient transport. Some authors have suggested that enhanced etiological assessment of dyspnea may support “treat-and-release” or “treat-and-refer” strategies in selected patients, potentially reducing certain short hospital admissions, provided that appropriate clinical selection and system safeguards are in place [17].

The agreement analysis between etiological categories derived from ICD-10 coding and categories inferred from LUS profiles demonstrated moderate concordance (κ = 0.36). This finding suggests that lung ultrasound may serve as an important adjunct to the diagnostic process, although it does not replace comprehensive clinical assessment. Notably, in a substantial proportion of cases categorized as “other” based on ICD-10 coding, LUS findings suggested a more specific etiological category. This observation may indicate that lung ultrasound has the potential to refine diagnostic classification in the prehospital setting.

An additional strength of the present study lies in its educational perspective. The distribution of observed sonographic features provides insight into which aspects of lung ultrasound may be particularly relevant in the training of paramedics and emergency medical personnel. Existing literature indicates that the A-profile (predominant A-lines with preserved pleural sliding) supports obstructive diagnoses and may assist in differentiating them from pulmonary edema. Conversely, diffuse bilateral B-lines with preserved pleural sliding represent a typical ultrasound pattern of pulmonary edema. Pulmonary consolidations and subpleural abnormalities are more frequently associated with pneumonia. Meta-analyses conducted in hospital settings demonstrate high sensitivity of lung ultrasound for pneumonia detection compared with chest radiography, although extrapolation of these findings to the prehospital environment requires caution [18]. Detection of pleural effusion represents another example of a sonographic finding with relatively straightforward interpretation, which makes it particularly valuable in ultrasound training programs [19,20].

From a broader systems perspective, the findings of this study suggest that implementation of lung ultrasound within emergency medical services may contribute to improved diagnostic precision and optimization of transport decisions in selected groups of patients presenting with dyspnea. Nevertheless, further research—particularly prospective and multicenter studies—is required to more precisely determine the clinical and system-level impact of lung ultrasound in prehospital care.

### Limitations of the Study

This study has several important limitations. First, the analysis was retrospective and relied on documentation from emergency medical services records, which may be subject to incomplete data or inaccuracies in ICD-10 coding. Second, the study was conducted in a single county in which ultrasound was systematically available in all EMS units. This may limit the generalizability of the findings to other emergency medical systems where ultrasound availability and training may differ.

Another limitation is that the decision to perform a lung ultrasound was made by the EMS team and was not randomized, which introduces the possibility of selection bias. Additional clinical factors that may influence transport decisions—such as overall patient condition, comorbidities, or system-level protocols—could not be fully controlled for in this analysis.

The exclusion of non-respiratory heart failure codes such as I50 was intended to preserve the respiratory focus of the cohort, although it may have limited capture of some patients with cardiogenic dyspnea coded primarily under circulatory diagnoses.

In the comparison with regional data, aggregated information from the National Emergency Medical Services Monitoring Centre was used. These data did not include information regarding ultrasound use or detailed clinical variables, which limits the ability to fully assess the causal relationship between ultrasound implementation and transport decisions at the regional level.

Finally, the agreement analysis between LUS findings and ICD-10 diagnostic categories relied on documentation-based diagnostic coding rather than final hospital diagnoses. As a result, ICD-10 codes may not always reflect the definitive diagnosis established after full hospital evaluation.

In addition, the availability of ultrasound devices and the level of operator training may vary substantially between EMS systems, as shown in previous reports on prehospital ultrasound implementation [16,17]. This may affect the external validity of the present findings.

The observed associations should not be interpreted as causal due to the retrospective design and potential selection bias.

## 5. Conclusions

Lung ultrasound appears to provide meaningful diagnostic support in the prehospital assessment of patients with dyspnea and respiratory failure. In this study, its use was associated with more etiology-oriented diagnostic coding and with characteristic sonographic patterns corresponding to inflammatory and cardiological categories. Moreover, the EMS system with systematically implemented ultrasound showed lower hospital transport rates for selected respiratory diagnostic groups compared with regional data. Further prospective multicenter studies are warranted.

## Figures and Tables

**Table 1:** ICD-10 codes included in the study and their clinical categorization.

Clinical Category	ICD-10 Code	Description
General/symptom-based	R06.0	Dyspnea
General/symptom-based	R06	Abnormalities of breathing
General/symptom-based	R06.8	Other specified breathing abnormalities
General/symptom-based	J96	Respiratory failure, not elsewhere classified
General/symptom-based	J96.1	Chronic respiratory failure
General/symptom-based	J96.0	Acute respiratory failure
General/symptom-based	J80	Acute respiratory distress syndrome
General/symptom-based	J00	Acute nasopharyngitis
General/symptom-based	J94	Other pleural conditions
General/symptom-based	J93	Pneumothorax
Inflammatory	J16	Pneumonia due to other infectious organisms
Inflammatory	J17	Pneumonia in diseases classified elsewhere
Inflammatory	J12	Viral pneumonia
Inflammatory	J12.8	Viral pneumonia due to other viruses
Inflammatory	J18.0	Pneumonia, unspecified
Inflammatory	J40	Bronchitis, not specified as acute or chronic
Inflammatory	J20	Acute bronchitis
Cardiological	J81	Pulmonary edema
Cardiological	J90	Pleural effusion
Obstructive	J44	Chronic obstructive pulmonary disease
Obstructive	J44.9	COPD, unspecified
Obstructive	J44.1	COPD with acute lower respiratory infection
Obstructive	J44.8	Other specified COPD
Obstructive	J45	Asthma
Obstructive	J45.9	Asthma, unspecified
Obstructive	J46	Status asthmaticus
Neoplastic	C34	Malignant neoplasm of bronchus and lung

**Table 2:** Mapping of lung ultrasound findings to clinical diagnostic categories.

Clinical Category	Typical LUS Findings	Clinical Interpretation
Cardiological	Diffuse bilateral B-lines ± pleural effusion	Suggestive of pulmonary edema/cardiogenic origin
Inflammatory	Consolidations, subpleural consolidations, focal B-lines	Suggestive of pneumonia or inflammatory pathology
Obstructive	A-profile (normal aeration), preserved lung sliding	Compatible with obstructive diseases (e.g., COPD, asthma)
Other	Pneumothorax signs (lung point, absent sliding) or mixed patterns	Non-specific or alternative pathology

**Table 3:** Characteristics of the study population.

Variable	N	%
Total EMS interventions included	353	100
Lung ultrasound performed	72	20.4
No lung ultrasound performed	278	78.8
Missing data on ultrasound performance	3	0.8
Transport to emergency department	239	67.7
No transport	114	32.3

**Table 4:** Clinical categories based on ICD-10 codes.

Clinical Category	N	%
General/symptom-based	182	51.6
Inflammatory	77	21.8
Obstructive	66	18.7
Cardiological	20	5.7
Other	7	2.0
Neoplastic	1	0.3

**Table 5:** Sonographic findings in patients who underwent lung ultrasound (*n* = 72).

Sonographic Finding	N	%
B-lines	43	61.4
Pulmonary consolidations	29	41.4
Subpleural consolidations (C-line type artefacts)	23	32.9
Pleural effusion	11	15.3
Pneumothorax signs (absent pleural sliding and/or lung point)	2	2.8
Normal aeration profile (A-profile)	16	22.2

**Table 6:** Impact of ultrasound and diagnostic precision.

Variable	LUS Performed	No LUS	RR (95% CI)
Targeted diagnosis (non-symptom ICD-10)	53/72 (73.6%)	111/278 (39.9%)	1.84 (1.51–2.25)
Specific primary ICD-10 diagnosis	41/72 (56.9%)	104/278 (37.4%)	1.52 (1.18–1.96)

**Table 7:** Comparison of hospital transport rates: study population vs. regional data.

Population	Transport to ED	Total Cases	%
Legionowo County	279	409	68.2
Masovian Voivodeship (excluding study county)	11,351	13,785	82.3

**Table 8:** Relative risk of hospital transport.

Comparison	RR	95% CI	*p*-Value
Legionowo vs. regional data	0.83	0.78–0.89	<0.001

**Table 9:** Selected ICD-10 transport differences.

ICD-10 Code	Study Population Transport	Regional Transport	RR
R06.0 Dyspnea	72.9%	88.2%	0.83
J44/J45/J46 Obstructive diseases	43.1%	67.0%	0.64

## Data Availability

The datasets generated and/or analyzed during the current study are available from the corresponding author on reasonable request.

## Supplementary Materials

The following supporting information can be downloaded at: https://www.mdpi.com/article/10.3390/diagnostics16091297/s1, Table S1: Aggregated regional EMS data from the Masovian Voivodeship (excluding Legionowo County) between 1 January and 30 June 2025. Table S2: Full dataset of EMS patients from Legionowo County, including assigned ICD-10 codes and corresponding lung ultrasound findings.
